# The Impact of COVID-19 Lockdown on Injuries in Saudi Arabia: Results From a Level-I Trauma Center

**DOI:** 10.3389/fpubh.2021.704294

**Published:** 2021-07-13

**Authors:** Faisal F. Hakeem, Saeed Mastour Alshahrani, Mohammed Al Ghobain, Ibrahim Albabtain, Omar Aldibasi, Suliman Alghnam

**Affiliations:** ^1^Department of Preventive Dental Sciences, Taibah University Dental College & Hospital, Madinah, Saudi Arabia; ^2^Basic Medical Sciences Department, College of Applied Medical Sciences, King Khalid University, Abha, Saudi Arabia; ^3^Department of Medicine, King Abdulaziz Medical City, Riyadh, Saudi Arabia; ^4^College of Medicine, King Saud Bin Abdulaziz University for Health Sciences, Riyadh, Saudi Arabia; ^5^King Abdullah International Medical Research Center, Riyadh, Saudi Arabia; ^6^Department of Surgery, King Abdulaziz Medical City, Riyadh, Saudi Arabia; ^7^Biostatistics and Bioinformatics, King Abdullah International Medical Research Center, Riyadh, Saudi Arabia; ^8^King Saud Bin Abdulaziz University for Health Sciences, Riyadh, Saudi Arabia; ^9^Population Health Department, King Abdullah International Medical Research Center, Riyadh, Saudi Arabia

**Keywords:** COVID-19, lockdown, trauma, injuries, epidemiology

## Abstract

**Background:** The COVID-19 pandemic has placed an enormous strain on global health. Due to precautionary measures, the epidemiology of health conditions may have been affected. Saudi Arabia imposed a lockdown order on March 25, 2020. This study investigated the impact of the pandemic lockdown on injuries in a level-I trauma center in King Abdulaziz Medical City, Riyadh, Saudi Arabia.

**Methods:** This retrospective study identified all injured patients seeking emergency care during the lockdown period (March 25–June 21, 2020) and a similar period in two previous year (March 25–June 21) 2018 and 2019. The collected data included patients' demographics, injury types, mechanisms, and health outcomes.

**Results:** Two hundred sixty nine injured patients sought emergency care during the lockdown, while 626 and 696 patients were treated in the same period of 2018 and 2019, respectively. There was a significant reduction in motor vehicle crashes (OR: 0.47; 95% CI: 0.31–0.73) and burns (OR: 0.24; 95% CI: 0.08–0.66), coupled with a significant increase in assault injuries (OR: 2.20; 95% CI: 1.30–3.74) in the lockdown period compared to 2019. Apart from the intensive care unit (ICU) admission and hospital length of stay, there were no differences between the two periods in the health outcomes. ICU admission was significantly reduced by 57% during the lockdown period (OR: 0.43; 95% CI: 0.22–0.83). Mechanisms of injuries were not significant predictors of deaths or ICU admission or both in the lockdown period.

**Conclusion:** The COVID-19 lockdown had a clear impact on the volume and mechanisms of injuries. The findings highlight that injury risk factors are modifiable and emphasize the importance of public health measures for preventing injuries and the significance of maintaining trauma services capacity during pandemics.

## Introduction

The COVID-19 pandemic has changed the global burden of disease (1). With the high spread of cases worldwide, the World Health Organization announced a “pandemic” status on March 11, 2020 (2). COVID-19 has placed a significant burden on the provision of healthcare and the sustainability of healthcare systems worldwide. Furthermore, morbidity and mortality due to COVID-19 have prompted drastic public health measures by governments and authorities to control the spread of the virus, including strict social distancing guidelines, prolonged school closure, reduced non-essential travels, and complete or partial lockdowns (3). Consequently, these measures reduced demand for healthcare systems (4).

Although applying such policies is likely to restrict the transmission of COVID-19, they can also impact the epidemiology of other health conditions, such as injuries, due to the constraints on daily life activities (5, 6). Globally, findings from multiple studies have shown an overall reduction in injuries, with no change in the type and severity during the pandemic (7). A study conducted in a single trauma center in the United States (US) found overall reductions in injury severity, mortality, and traffic-related injuries during the lockdown compared to a similar period in 2019. The study found significant reductions in pedestrian, motorcycle, and bicycle injuries in the lockdown period relative to 2019. Nevertheless, there were no significant reductions in motor vehicle crashes (8). Another US study found no significant decrease in crashes that resulted in severe or fatal injuries following the lockdown. The authors found that the lockdown only decreased traffic crashes resulting in non-serious or no injuries (9). A study conducted in Italy that investigated wrist and hand injuries found a significant reduction in traffic crashes during the lockdown period than in 2019 (10). Similarly, a decrease in trauma has been observed during the COVID-19 pandemic in Netherlands, New Zealand, Australia, South Africa, and Austria (11–16).

The government of Saudi Arabia took a series of measures in response to the COVID-19 pandemic following the discovery of the first COVID-19 case on March 2, 2020. The first three major measures were the suspension of Umrah pilgrimage, suspension of all schools and universities, and suspension of international flights. These measures were followed by imposing a partial lockdown and restricting movement between regions on March 25. Next, the country imposed a full nationwide lockdown from April 6 until May 28. After that, the lockdown was partially lifted from most of the cities, the travel between regions and domestic flights were permitted, and retail stores, shopping centers, and restaurants were reopened. On June 21, the lockdown was completely lifted from all regions of Saudi Arabia (17).

Road traffic injuries are a major public health concern in Saudi Arabia (18). They are the third leading cause of death and the leading cause of years of life lost (YLLs) (19). Around one-fifth of hospital beds in the country are regularly occupied by victims of traffic crashes, who also account for most trauma death cases (20). Thus, road traffic injuries pose a serious risk to the health and well-being of the Saudi population.

Even though global literature has examined trauma epidemiology during the COVID-19 pandemic, the impact of measures taken in the COVID-19 pandemic will likely vary across countries. Exploring the impact of COVID-19 lockdown on trauma in Saudi Arabia, where there is a huge burden of injuries, can enhance our understanding of potential preventive measures of injuries. In addition, evaluating the impact of lockdown on injuries can inform policies aimed to promote more sustainable and secure transport systems for the post-COVID-19 era. Therefore, we aim to investigate the impact of the pandemic lockdown on injuries in Riyadh city using data from a trauma registry. The study's objective is to compare the frequency, type, clinical characteristics, mechanism of injuries, and outcomes of injured cases during the lockdown compared to a similar period in the previous 2 years.

## Methods

This is a retrospective study of all trauma visits to King Abdulaziz Medical City (KAMC) in Riyadh. KAMC, which is located in the eastern part of Riyadh, has a bed capacity of 1,501. Although the catchment area of KAMC is the eastern part of Riyadh; some patients are transferred from other hospitals because KAMC is an advanced trauma center. KAMC meets the criteria for a level 1 trauma center and a designated site to provide courses in Advanced Trauma Life Support (ATLS) (21).

The study focused on 3 years of data to compare trauma patterns and outcomes (between March 25–June 21, 2020, March 25–June 21, 2019 and March 25–June 21, 2018). All patients with trauma presented to the emergency department during the two periods were included in this study. Patients were identified from the KAMC trauma registry (18). This registry was initiated in 2016 and included any patient that is admitted to the hospital following any physical injury. In addition, patients who died either prior to arrival or after medical care are also captured. Three trained coordinators identify patients from the electronic medical record system using the International Classification of Disease (ICD) 10th version and then enter their information into the registry. The study used a predesigned data collection sheet to collect data from the trauma registry. The IRB approval was obtained from KAIMRC Institutional Review Board (NRC21R/027/01).

The variables collected include patients' demographics, injury type, mechanism, and health outcomes such as death, intensive care unit (ICU) admission, and hospital length of stay. The registry employs quality checks every year by taking a random sample of 100 records and validating them. The primary outcome variables included in the study were: mortality, Injury Severity Score (ISS), admission to the ICU and disability. We captured disability based on the functional independence measure (FIM), which was evaluated at discharge from the hospital (22).

### Statistical Analysis

SPSS version 21.0 software (SPSS Inc., Chicago, IL, USA) was used to perform all analyses. Frequency and percentage were used to report categorical variables and mean and standard deviation (SD), or median and interquartile range (IQR) to report continuous variables. To assess the bivariate relationships between the year of emergency department (ED) admission and several variables of interest including demographic and clinical characteristics, Chi-squared test, for categorical variables, and Non-parametric Kruskal Wallis test for continuous variables. Analysis of Variance (ANOVA) was used to test for age distribution across the years of ED admission.

Multivariable logistic regression models were constructed to assess how the year of ED admission—the independent variable (IV)—can predict the dependent variables (DVs), including mechanism of injuries (Yes/No), mortality (Yes/No), ICU admission (Yes/No), ISS (mild/moderate/severe), disability score (totally independent/partially or totally dependent), and hospital length of stay (continuous). Covariates included in the logistic regression models were age, gender, occupation, marital status and comorbidities including diabetes mellitus, hypertension, respiratory diseases, dyslipidemia, depression, and smoking status as well as prehospital transportation and vital signs upon arrival into ED. Length of stay was modeled using multivariable negative binomial regression adjusting for the same covariate. Finally, a stratified analysis by the year of ED admission was conducted to investigate the influence of each mechanism of injuries—as independent variables (IVs)—in predicting the death or ICU admission or both—as dependent variable (DV)—during the lockdown period in 2020 compared with the similar periods of 2018 and 2019. Thus, a subgroup analysis among those with injuries resulting in either death and ICU admission or both were conducted. That is, either death and ICU admission or both were evaluated as a single variable to increase the test power of evaluating adverse outcomes. Model assumptions including multicollinearity and linearity were investigated. Also, overdispersion was assessed for negative binomial regression. No violation of the assumptions was observed. A *P*-value < 0.05 was considered significant in all analyses.

## Results

### Demographic and Clinical Characteristics

A total of 1,591 injured patients presented to ED and were included in this study. Of those, 626 (39.3%) were in 2018, 696 (43.7%) were in 2019, and 269 (16.9%) were in 2020. The distributions of age and gender were approximately similar for those who were admitted in 2019 and 2020. That is, the mean age and SD of the patients admitted were 32.1 (23.5), 31.9 (23.4) and 32.7 (23.5) for 2018, 2019, and 2020, respectively. Approximately two-thirds of injured patients were males in both years (71.9, 71.6 and 72.9% for 2018, 2019, and 2020, respectively). In addition, less injuries among unemployed were observed in 2020 (37.5%) as compared to 2018 (57.5%) and 2019 (48.4%). Also, injuries among children were less in 2020 (16.7%) as compared to 2018 (25.6%) and 2019 (17.8%). In addition, comorbidities including diabetes, hypertension, respiratory diseases, dyslipidemia, depression, and smoking status appeared to be almost similar across the 3 years. Transportation mode to the hospital was significantly different between 2018, 2019, and 2020 (ambulance: 24.3 vs. 25.7 vs. 30.5%; Private: 38.8 vs. 26 vs. 30.9%; *P-value* < *0.001*). The Glasgow Coma Scale and other vital signs, including systolic blood pressure, heart rate, oxygen saturation, and respiratory rate measured upon ED admission were not significantly different between 2018, 2019, and 2020 (Table 1).

**Table 1:** Demographic and clinical characteristics of patients presented in ED with injuries.

		**Year of ED admission**			
**Variable**		**2018**	**2019**	**2020**	
		***N* = 626**	***N* = 696**	***N* = 269**	***P*-value**
Age, mean (SD)		32.1 (23.5)	31.8 (23.4)	32.7 (23.5)	0.888^a^
Age groups, *n* (%)	<18	200 (31.9)	215 (30.9)	77 (28.6)	0.925^b^
	18–24	73 (11.7)	98 (14.1)	37 (13.8)	
	25–34	121 (19.3)	118 (17.0)	52 (19.3)	
	35–44	73 (11.7)	87 (12.5)	33 (12.3)	
	45–54	39 (6.2)	50 (7.2)	15 (5.6)	
	55–64	38 (6.1)	42 (6.0)	21 (7.8)	
	≥65	82 (13.1)	86 (12.4)	34 (12.6)	
Gender, *n* (%)	Male	450 (71.9)	498 (71.6)	196 (72.9)	0.921^b^
	Female	176 (28.1)	198 (28.4)	73 (27.1)	
Occupation, *n* (%)	Employed	94 (15.0)	76 (10.9)	32 (11.9)	<0.001^b^
	Unemployed	360 (57.5)	337 (48.4)	101 (37.5)	
	Not recorded/not applicable	172 (27.5)	283 (40.7)	136 (50.6)	
Marital status, *n* (%)	Married	189 (30.2)	226 (32.5)	94 (34.9)	0.004^b^
	Unmarried	277 (44.2)	346 (49.7)	130 (48.3)	
	Child	160 (25.6)	124 (17.8)	45 (16.7)	
Diabetes mellitus, *n* (%)	Yes	74 (11.8)	83 (11.9)	34 (12.6)	0.939^b^
	No	552 (88.2)	613 (88.1)	235 (87.4)	
Hypertension, *n* (%)	Yes	78 (12.5)	85 (12.2)	40 (14.9)	0.518^b^
	No	548 (87.5)	611 (87.8)	229 (85.1)	
Respiratory disease, *n* (%)	Yes	20 (3.2)	16 (2.3)	8 (3.0)	0.596^b^
	No	606 (96.8)	680 (97.7)	261 (97.0)	
Dyslipidemia, *n* (%)	Yes	23 (3.7)	30 (4.3)	12 (4.5)	0.796^b^
	No	603 (96.3)	666 (95.7)	257 (95.5)	
Depression, *n* (%)	Yes	4 (0.6)	10 (1.4)	2 (0.7)	0.348^b^
	No	622 (99.4)	686 (98.6)	267 (99.3)	
Current smoker, *n* (%)	Yes	33 (5.3)	46 (6.6)	14 (5.2)	0.519^b^
	No	593 (94.7)	650 (93.4)	255 (94.8)	
Transport mode to hospital, *n* (%)	Ambulance	152 (24.3)	179 (25.7)	82 (30.5)	<0.001^b^
	Private	243 (38.8)	181 (26.0)	83 (30.9)	
	Other	138 (22.0)	195 (28.0)	52 (19.3)	
	Unknown	93 (14.9)	141 (20.3)	52 (19.3)	
Glasgow Coma Scale (GCS), *n* (%)	Mild (<9)	42 (6.8)	45 (6.5)	13 (4.8)	0.767^b^
	Moderate (9–13)	24 (3.9)	32 (4.6)	13 (4.8)	
	Severe (>13)	554 (89.4)	613 (88.8)	243 (90.3)	
	*Missing*	*6*	*6*	*0*	
Systolic Blood Pressure (mmHg), median (IQR)^c^		120 (109–135)	121 (109–135)	124 (109–140)	0.302^d^
Heart rate (beats per minutes), median (IQR)^c^		94 (82–110)	95 (82–112)	93 (80–110.5)	0.953^d^
Oxygen saturation (%), median (IQR)^c^		98 (97–99	98 (97–100)	98 (97–99)	0.620^d^
Respiratory rate (breaths per minute), median (IQR)^c^		20 (20–25)	20 (20–24)	20 (20–23.5)	0.389^d^

a *Derived from Analysis of Variance (ANOVA) Fisher test*.

b *Derived from Chi-square test*.

c *IQR, Interquartile Range*.

d *Derived from Non-Parametric Kruskal Wallis test*.

### Mechanism of Injury

**Figure 1:**
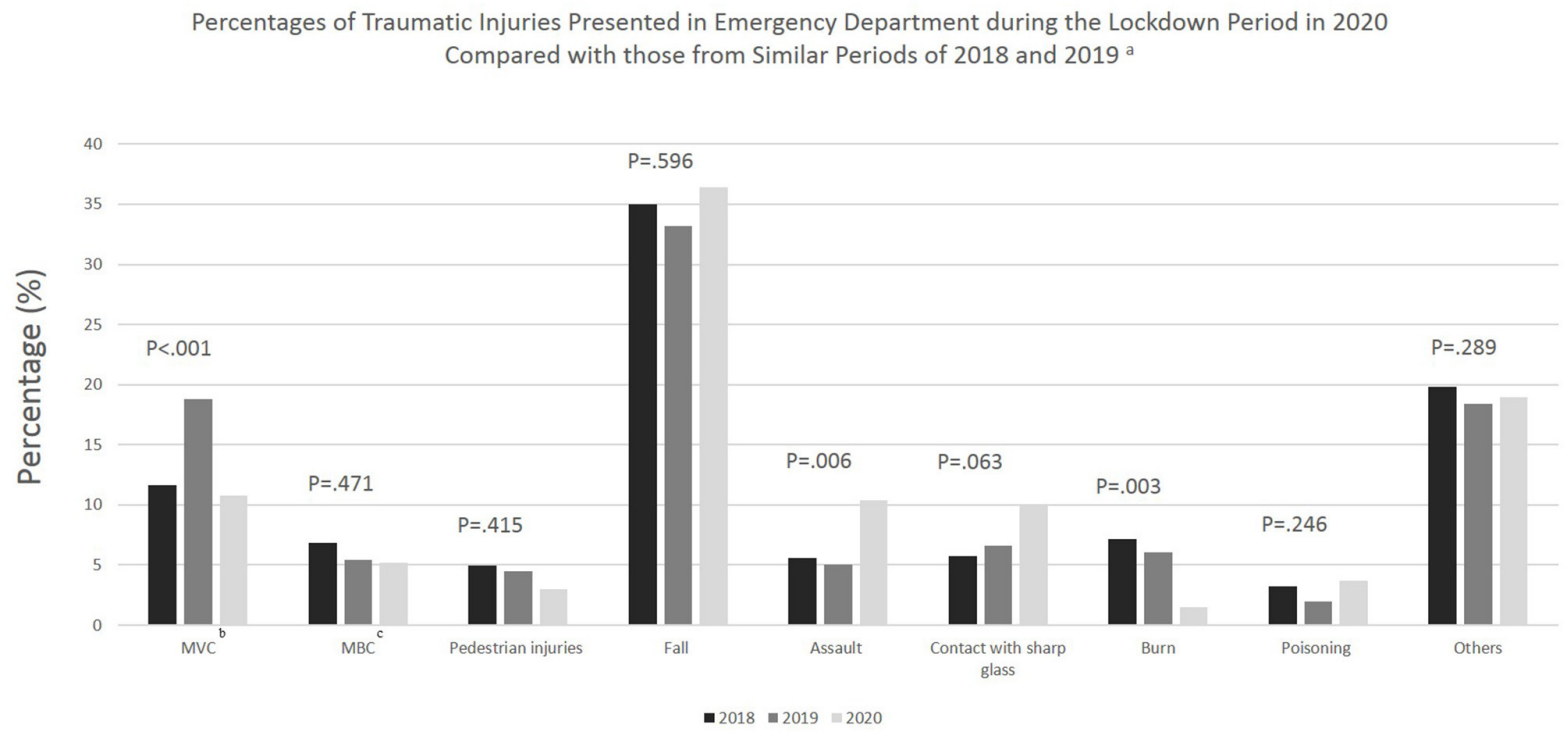
Percentages of traumatic injuries presented in emergency department during the lockdown period in 2020 compared with those from similar periods of 2018 and 2019. ^a^*P*-value (P) are produced from Chi-squared test. *P*-value is considered significant when <0.05. ^b^MVC, Motor-vehicle crashes. ^c^MBC, Motorcycle and bicycle crashes.

Regarding the differences in injury mechanism, results from multivariable logistic regression show that the odds of an injury mechanism being related to a motor vehicle crash was 53% lower in 2020 (during COVID) compared to 2019 (pre-COVID) (OR: 0.47; 95% CI: 0.31–0.73). Also, the odds of injuries caused by burns were reduced by 76% in 2020 as compared with 2019 (OR: 0.24; 95% CI: 0.08–0.67). On the other hand, the odds of an injury mechanism being related to assault was over 2-fold higher in 2020 compared to 2019 (OR: 2.20; 95% CI: 1.30–3.74). Moreover, there was a borderline significant increase in injuries due to contact with sharp glass from 2019 and 2020 (OR: 1.65; 95% CI: 1.00–2.77) (Table 2 and Figure 1).

**Table 2:** The association of the lockdown period of 2020 and the similar periods of 2018 and 2019 with several mechanisms of injury presented as odd ratios (OR) and 95% confidence interval (CI)^a^.

		**Year of ED admission**^*******b*******^		
**Mechanism**^*******c*******^, ***n*** **(%)**		**2018**	**2019**	**2020**
		***N* = 626**	***N* = 696**	***N* = 269**
MVC^d^		73 (11.7)	131 (18.8)	29 (10.8)
	OR (95% CI)	0.64 (0.7–0.88)	Ref	0.47 (0.31–0.73)
MBC^e^		43 (6.9)	38 (5.5)	14 (5.2)
	OR (95% CI)	1.20 (0.75–1.90)	Ref	0.95 (0.50–1.79)
PI^f^		31 (5.0)	31 (4.5)	8 (3.0)
	OR (95% CI)	1.07 (0.64–1.82)	Ref	0.66 (0.30–1.47)
Fall		219 (35.0)	231 (33.2)	98 (36.4)
	OR (95% CI)	1.04 (0.81–1.34)	Ref	1.19 (0.87–1.64)
Assault		35 (5.6)	35 (5.0)	28 (10.4)
	OR (95% CI)	1.21 (0.74–1.98)	Ref	2.20 (1.30–3.74)
Contact with sharp glass		36 (5.8)	46 (6.6)	27 (10.0)
	OR (95% CI)	0.86 (0.54–1.37)	Ref	1.65 (1.00–2.77)
Burn		45 (7.2)	42 (6.0)	4 (1.5)
	OR (95% CI)	1.23 (0.79–1.93)	Ref	0.24 (0.08–0.67)
Poisoning		20 (3.2)	14 (2.0)	10 (3.7)
	OR (95% CI)	1.98 (0.94–4.18)	Ref	2.37 (0.98–5.72)
Others		124 (19.8)	128 (18.4)	51 (19.0)
	OR (95% CI)	1.23 (0.89–1.69)	Ref	0.90 (0.58–1.39)

a *Derived from multivariable binary logistic regression adjusting for demographic factors including age, gender, occupation, and marital status and comorbidities including diabetes mellitus, hypertension, respiratory diseases, dyslipidemia, depression, and smoking status*.

b *Independent Variable (IV)*.

c *Dependent Variables (DVs)*.

d *MVC, Motor-vehicle crashes*.

e *MBC, Motorcycle and bicycle crashes*.

f *PI, Pedestrian injuries*.

### Outcomes

The major outcomes of interest in this study, including death, injury severity, and disability were not significantly predicted by the year of admission. However, the odds of injury-related ICU admission was 57% lower during lockdown period in 2020 compared to the similar period in 2019 (OR: 0.43; 95% CI: 0.22–0.83). In addition, results from multivariable negative binomial regression indicated that those who were admitted during the lockdown period in 2020 had significantly lower days of stay in the hospital (−0.35 log days) compared to those who were admitted in the similar period in 2019 (Table 3).

**Table 3:** Outcomes: Death, ICU admission, injury severity, disability and hospital length of stay^a^.

		**Year of ED admission**^*******b*******^		
**Outcome^***c***^**		**2018**	**2019**	**2020**
		***N* = 626**	***N* = 696**	***N* = 269**
Death, *n* (%)		22 (3.5)	13 (1.9)	5 (1.9)
	OR (95% CI)^d^	2.11 (0.84–5.32)	Ref	1.09 (0.25–4.85)
ICU admission, *n* (%)		120 (19.4)	127 (18.2)	33 (12.3)
	OR (95% CI)^d^	0.99 (0.67–1.46)	Ref	0.43 (0.22–0.83)
Injury severity score (ISS), *n* (%)			
Mild (ISS <9)		360 (59.8)	395 (60.2)	147 (58.1)
Moderate (ISS (9–15))		145 (24.1)	159 (24.2)	68 (26.9)
Severe (ISS>15)		97 (16.1)	102 (15.5)	38 (15.0)
*Missing*		*24*	*40*	*16*
	OR (95% CI)^e^	1.06 (0.81–1.40)	Ref	0.90 (0.61–1.32)
Pre-existent disability score, *n* (%)			
12 (Total independence)		374 (85.8)	464 (94.9)	189 (94.0)
<12 (Partial or total dependent)		62 (14.2)	25 (5.1)	12 (6.0)
*Missing*		*190*	*207*	*68*
	OR (95% CI)^d^	3.28 (1.74–6.19)	Ref	0.79 (0.28–2.24)
Discharge disability score				
12 (Total independence)		147 (34.2)	143 (29.7)	47 (23.5)
<12 (Partial or total dependent)		283 (65.8)	339 (70.3)	153 (76.5)
*Missing*		*196*	*214*	*69*
	OR (95% CI) ^d^	1.25 (0.89–1.77)	Ref	1.44 (0.90–2.31)
Disability score difference				
Deterioration (Discharged with increased disability)		226 (52.6)	314 (65.1)	141 (70.5)
No deterioration		200 (46.5)	168 (34.9)	59 (29.5)
*Missing*		*200*	*214*	*79*
	OR (95% CI)^d^	0.87 (0.63–1.20)	Ref	1.47 (0.94–2.28)
Hospital length of stay (days), median (IQR)^f^		3 (1–9)	4 (1–10)	3 (1–8)
	β (95% CI)^g^	−0.21 (−0.63 to −0.04)	Ref	−0.35 (−0.59 to −0.10)

a *All models are adjusted for demographic factors including age, gender, occupation, and marital status and comorbidities including diabetes mellitus, hypertension, respiratory diseases, dyslipidemia, depression, and smoking status as well as prehospital transportation and vital signs upon arrival into ED*.

b *Independent Variable (IV)*.

c *Dependent Variables (DVs)*.

d *Derived from multivariable binary logistic regression*.

e *Derived from multivariable ordinal logistic regression. Severe injuries (ISS>15) is the reference group (i.e., the value we were interested to predict in our model)*.

f *IQR, Interquartile Range*.

g *Derived from multivariable negative binomial regression*.

### Causes of Death or ICU Admission or Both

The number of deaths or ICU admission or both in 2018, 2019, and 2020 were 123, 127, and 34, respectively. Results from stratified analysis by the year of ED admission show changes in the predictors of death or ICU admission or both across the years of admission. That is, major predictors of death or ICU admission or both in 2018 were motor-vehicle injuries (OR: 4.18; 95% CI: 1.06–16.47), motorcycle and bicycle injuries (OR: 9.01; 95% CI: 2.18–37.22), pedestrian injuries (OR: 11.85; 95% CI: 2.69–52.17), assaults (OR: 3.86; 95% CI: 1.04–14.32) and burns (OR: 9.17; 95% CI: 2.47–30.04). Regarding 2019, major predictors of death or ICU admission or both were pedestrian injuries (OR: 4.13; 95% CI: 1.18–14.51), burns (OR: 9.67; 95% CI: 3.08–30.31) and poisoning (OR: 13.84; 95% CI: 3.07–62.37). In 2020, however, none of the previously mentioned mechanisms of injuries were significant predictors of deaths or ICU admission or both (Table 4).

**Table 4:** Causes (significant predictors) of death or ICU admission or both^a^.

**Causes^***b***^, *n* (%)**	**Death or ICU admission or both**^*******c*******^ **stratified by year of ED admission**					
	**2018**		**2019**		**2020**	
	***n* = 123**	**OR (95% CI)^***d***^**	***n* = 127**	**OR (95% CI)^***d***^**	***n* = 34**	**OR (95% CI)^***d***^**
MVC^e^	23 (18.7)	4.18 (1.06–16.47)	31 (24.4)	1.83 (0.62–5.36)	5 (14.7)	2.14 (0.38–12.11)
MBC^f^	22 (17.9)	9.01 (2.18–37.22)	12 (9.4)	2.65 (0.78–9.01)	2 (5.9)	1.98 (0.24–16.3)
PI^g^	17 (13.8)	11.85 (2.69–52.17)	11 (8.7)	4.13 (1.18–14.51)	3 (8.8)	7.81 (0.95–64.12)
Fall	18 (14.6)	1.59 (0.40–6.31)	29 (22.8)	1.51 (0.52–4.38)	7 (20.6)	2.41 (0.45–12.96)
Assault	9 (7.3)	3.86 (1.04–14.32)	5 (3.9)	0.94 (0.25–3.62)	6 (17.6)	4.47 (0.88–22.76)
Burn	14 (11.4)	9.17 (2.47–30.04)	15 (11.8)	9.67 (3.08–30.31)	1 (2.9)	10.47 (0.58–188.12)
Poisoning	3 (2.4)	2.82 (0.46–17.31)	6 (4.7)	13.84 (3.07–62.37)	2 (5.9)	5.85 (0.8–42.89)
Others	17 (13.8)	3.71 (0.95–14.46)	18 (14.2)	1.65 (0.53–5.13)	8 (23.5)	4.16 (0.78–22.14)

a *All models in this table were run separately for each year (i.e., data were stratified by the year of ED admission) to investigate the influence of each cause in predicting death or ICU admission or both during the lockdown period in 2020 compared with the similar periods of 2018 and 2019*.

b *Independent Variables (IVs)*.

c *Dependent Variable (DV)*.

d *Derived from multivariable binary logistic regression adjusting for age, gender and prehospital transportation*.

e *MVC, Motor-vehicle crashes*.

f *MBC, Motorcycle and bicycle crashes*.

g *PI, Pedestrian Injuries*.

## Discussion

The present study was designed to determine the impact of the pandemic lockdown on trauma in Saudi Arabia. We found that Covid-19 had an impact on trauma epidemiology, but perhaps the most striking finding is the significant reduction of trauma admission in general and motor vehicle crashes specifically. Surprisingly, the admission year did not predict death, injury severity score and disability. However, ICU admission and hospital length of stay were significantly reduced.

One of the main findings of this study is the clear reduction of injured patients presented to ED by 61.3 and 57% during the COVID-19 pandemic compared to the same period in 2019 and 2018, respectively. Demographic factors including age, gender, marital status, and comorbidities were similar between the pandemic year and the two previous periods. Our findings were not surprising and can be explained by the restriction of the movement during the lockdown period in Saudi Arabia, and they are consistent with similar reports from many parts of the world. For example, a study conducted in a level 1 Trauma Center in the US found a similar pattern (23). Furthermore, the percentage of reduction in trauma admission found in the present study is comparable to the estimates found in the literature. A recent scoping review found that the global reduction of trauma admissions during COVID-19 ranges between 20.3 and 84.6% compared to previous control periods (7). The most plausible explanation for such an observation is the public's adherence to lockdown and societal distancing principles.

Furthermore, we observed changes in the transportation mode, in particular an increase in using private vehicles for transportation to the hospital during the COVID-19 pandemic compared to the same period in 2020. This is in line with a previous study conducted in a trauma center in the US (8). A possible explanation for this change could be related to the fact that private cars are more acceptable in the Saudi community, and most of the trauma patients are living not far distance from the hospital. Moreover, there is a possibility that the emergency medical services (EMS) were overwhelmed with COVID-19 cases, causing a delay in transportation (24), which might lead to more people deciding not to wait and drive to the hospital themselves. An alternative explanation could be the increase of assault injuries and poisoning, which usually results in superficial injuries and is more likely to be transported using private vehicles. However, it should be highlighted that a previous local study conducted in Saudi Arabia demonstrated that over half of trauma patients arrived at the hospital via private transportation (25). Furthermore, consistent with the literature, the present study found that admission vital signs and comorbidities were similar between the COVID-19 pandemic period and the same periods in the previous 2 years (8).

We observed a general decrease in motor vehicle crashes and burns in terms of the mechanisms of injury in the COVID-19 pandemic. This is coupled with a significant increase in assault injuries and contact with sharp glass from 2019 and 2020. In accordance with these findings, previous studies have demonstrated similar trends in the etiology of injuries during the COVID-19 pandemic. A decrease in traffic-related injuries was observed in studies conducted in the US (23), the UK (26), Europe (27, 28), Australia (13, 29), and South Africa (14, 15). The present study's findings also accord with earlier observations from different studies, which showed an increase in assault injuries during the COVID-19 pandemic (30, 31). The decrease in traffic-related injuries and motor vehicle crashes reflects the drop in travel and the presence of fewer vehicles on the road due to the implementation of movement restrictions, lockdown measures, working from home policies, and the closure of schools.

On the other hand, the increase in assault injuries might be related to domestic violence, stress, depression, anxiety and the economic crises related to lockdown (32–34). In this study, we observed an increase in the proportion of falls during the lockdown compared to a similar period in 2019 and 2018 (36.4 vs. 33.2, and 35%, respectively). However, this increase was not statically significant. A scoping review on trauma during the pandemic found that falls from 2 meters, which is usually encountered by older adults, and falls from heights, which is common among children have increased during the pandemic (7). Moreover, we did not observe significant changes in foreign body, and poisoning between 2020 and 2019. A possible explanation for this might be that these injuries usually occur indoor. Thus, the exposure to risk factors did not decline.

Surprisingly, the admission year only predicted ICU admission and hospital length of stay. The admission year did not predict the other major outcomes, including death, injury severity score and disability. Previous studies found no differences in in-hospital outcomes, including ISS, death, ICU admission, and hospital length of stay between the pandemic year and previous periods (30, 35, 36). It should be highlighted that some studies which analyzed the whole pandemic year have observed a decrease in traffic death during the lockdown period, however, these changes were not sustained during the whole pandemic year (37, 38). These findings have significant implications for the management and workforce planning of trauma services during future pandemics or disasters. It emphasizes the value of sustaining adequately resourced trauma facilities and updating emergency preparedness plans for the post-COVID-19 era.

Even though there were no differences in major outcomes of the study apart from the ICU admission and hospital length of stay, we observed that motor-vehicle crashes, motorcycle and bicycle injuries, pedestrian injuries, assaults and burns were strong predictors of combined ICU or death outcome in 2018. On the other hand, in 2019, pedestrian injuries, assaults, burns and poisoning were strong predictors of combined ICU or death outcome. However, In the lockdown period, there was no significant predictors of the combined outcome. These findings provide some support for the conceptual premise that traumatic-related injuries are preventable. Clearly, public health measures had an impact on the number and mechanisms of traumatic injury seen. Therefore, further governmental public health measures are crucial for alleviating the burden of traumatic-related injuries in the post-COVID-19 era.

Some limitations need to be addressed regarding the present study. First, the study was conducted using data from a single-center, and thus the findings might not be generalizable to different cities and regions in Saudi Arabia. Second, as this is a retrospective study and inherits limitations may affect our results. Despite these limitations, this is the first study to assess the impact of governmental public health measures on traumatic injuries in Saudi Arabia. The study certainly adds to our understanding of the impact of COVID-19 lockdown on injuries. Further work needs to be done to establish whether the findings are generalizable to the whole country of Saudi Arabia. A national multicenter study is warranted to confirm our findings and further assess the impact of government public health measures on traumatic injuries epidemiology and outcomes to inform policies aiming to prevent traumatic injuries in the future. Future studies should also examine whether the observed changes and trends will reverse when the pandemic is over.

In conclusion, the COVID-19 pandemic and the lockdown had a clear impact on the volume and mechanisms of injuries. There was a clear reduction in the number of trauma cases, a significant decrease in motor vehicle crashes and burns, and a significant increase in assault injuries. The study's findings highlight the importance of governmental public health measures for preventing traumatic-related injuries and the significance of maintaining trauma services capacity and workforce during future pandemics and disasters.

## Data Availability Statement

All datasets generated for this study are available from the corresponding author upon reasonable request.

## Ethics Statement

The studies involving human participants were reviewed and approved by King Abdullah International Medical Research Center Institutional Review Board. Written informed consent to participate in this study was provided by the participants' legal guardian/next of kin.

## Author Contributions

SA, IA, OA, and FH conceived the research question and study design. IA and SA facilitated data collection and wrote the method section. SMA analyzed the data and wrote the results section. FH wrote the introduction and discussion section. OA, MA, and IA interpreted the study findings and modified the analyses as needed. All authors reviewed the final manuscript and provided input in improving the draft.

## Conflict of Interest

The authors declare that the research was conducted in the absence of any commercial or financial relationships that could be construed as a potential conflict of interest.
